# The prevalence of primary headache disorders in Ethiopia

**DOI:** 10.1186/s10194-016-0704-z

**Published:** 2016-12-07

**Authors:** Mehila Zebenigus, Redda Tekle-Haimanot, Dawit K. Worku, Hallie Thomas, Timothy J. Steiner

**Affiliations:** 1 Department of Neurology, School of Medicine, College of Health Science, Addis Ababa University, Addis Ababa, Ethiopia; 2 Department of Internal Medicine, School of Medicine, College of Health Science, Addis Ababa University, Addis Ababa, Ethiopia; 3 Department of Internal Medicine, Bahir Dar University, Bahir Dar, Ethiopia; 4 Department of Neuroscience, Norwegian University of Science and Technology (NTNU), Edvard Griegs Gate, NO-7491 Trondheim, Norway; 5 Division of Brain Sciences, Imperial College London, London, UK

**Keywords:** Epidemiology, Prevalence, Headache, Migraine, Tension-type headache, Medication-overuse headache, Ethiopia, Sub-Saharan Africa, Global Campaign against Headache

## Abstract

**Background:**

Knowledge of the epidemiology of primary headache disorders in sub-Saharan Africa (SSA) remains very limited. We performed a population-based survey in rural and urban areas of Ethiopia, using methods similar to those of an earlier study in Zambia and tested in multiple other countries by *Lifting The Burden*.

**Methods:**

In a cross-sectional survey we visited households unannounced in four regions of Ethiopia: the mostly urban populations in Addis Ababa and its environs and rural populations of selected districts in Oromia, Amhara and South Nations Nationalities and People’s Regions States (SNNPRS). We used cluster-randomized sampling: within clusters we randomly selected households, and one adult member (18–65 years old) of each household. The HARDSHIP structured questionnaire, translated into the local languages, was administered face-to-face by trained interviewers. Demographic enquiry was followed by diagnostic questions based on ICHD-II criteria.

**Results:**

From 2,528 households approached, 2,385 of 2,391 eligible members (1,064 [44.7%] male, 596 [25.0%] urban) consented to interview (participating proportion 99.8%). Headache in the preceding year was reported by 1,071 participants (44.9% [95% CI: 42.4–46.3]; males 37.7%, females 49.9%), and headache yesterday by 170 (7.1% [6.2–8.2]; males 45 [4.1%], females 125 [9.2%]). Adjusted for gender, age and habitation (urban/rural), 1-year prevalence of migraine was 17.7%, of tension-type headache (TTH) 20.6%, of all headache on ≥15 days/month 3.2%, and of probable medication-overuse headache (pMOH) 0.7%. The adjusted prevalence of headache yesterday was 6.4%. Very few cases (1.6%) were unclassifiable. All headache disorders were more common in females. TTH was less common in urban areas (OR: 0.3; *p* < 0.0001), but pMOH was very strongly associated (OR: 6.1; *p* < 0.0001) with urban dwelling. Education was negatively associated with migraine (OR: 0.5–0.7; *p* < 0.05) but (at university level) positively with pMOH (OR: 2.9; *p* = 0.067). Income above ETB 500/month showed similar associations: negatively with migraine (OR: 0.8; *p* = 0.035), positively with pMOH (OR: 2.1; *p* = 0.164).

**Conclusions:**

Findings for migraine and TTH in Ethiopia were quite similar to those from Zambia, another SSA country; pMOH was much less prevalent but, as in Zambia, essentially an urban problem. Primary headache disorders are at least as prevalent in SSA as in high-income western countries.

## Background

Knowledge of headache prevalence and headache-attributed burden is improving worldwide. Better methodology is being applied to population-based studies [1, 2] and a number of national surveys have been completed within the Global Campaign against Headache [3, 4] in world regions where information was lacking. It is now recognised that headache disorders, especially tension-type headache (TTH) and migraine, are among the most common medical conditions [1, 2] and collectively the third highest cause of years of life lost to disability (YLDs) [5, 6].

Sub-Saharan Africa (SSA) has been one of the large geographical areas devoid of data. The few studies of headache prevalence, including one in Ethiopian textile mill workers [7], have mostly been in select sub-populations rather than population-based [7–15], with estimates varying considerably but generally far lower than global means [16]. The consequent perception that headache disorders were relatively uncommon in SSA, and not of significant public-health concern, was reversed after the completion of a survey in Zambia [17, 18]. This study, supported by *Lifting The Burden* (LTB), a UK-registered non-governmental organization conducting the Global Campaign in official relations with the World Health Organization (WHO) [19], reported highly prevalent migraine (22.9%), TTH (22.8%) and probable medication-overuse headache (pMOH) (7.1%) [17]. These were associated with heavy disability burden and lost productivity, calling for action and recognition within public health policy [18].

This study in Ethiopia, also supported by LTB as a project within the Global Campaign against Headache, used very similar methods to the survey in Zambia. Its aims were to add to knowledge regarding primary headache disorders in SSA and discover whether the findings in Zambia would be replicated. The purposes were to inform health policy-makers in Ethiopia and to inform future global estimates undertaken by the Global Burden of Disease project (GBD) [20]. Here we report 1-year prevalences of headache, migraine, TTH, all causes of headache on ≥15 days/month and pMOH, and point (1-day) prevalence of headache (“headache yesterday”). Disability and other aspects of burden attributable to these disorders will be described elsewhere.

## Methods

### Ethics

Approvals were obtained from the Ethics Review Committee of the Department of Neurology, and the Institutional Review Board and Research and Publication Committee of the College of Health Sciences of Addis Ababa University, and from the Regional Health Bureau of each Region where the survey was undertaken.

Informed consent was obtained from participants before enrolment. Personal data were anonymised during analysis and dissemination.

### Study design

This was a cross-sectional questionnaire-based survey of adults aged 18-65 years. It was designed in accordance with published guidelines [1]. We selected and engaged with participants by calling at households unannounced. We used cluster-randomized sampling, randomly selecting households within clusters and one adult member of each biologically-unrelated family within a household.

### Survey sites

The main study was conducted in four Regions of Ethiopia: the mostly urban populations in Addis Ababa and its environs and rural populations of selected districts from Oromia, Amhara and South Nations Nationalities and People’s Regions States (SNNPRS).

The population distribution of Ethiopia was 17% urban, 83% rural (2011 data [21]). In order to have sufficient numbers for urban/rural comparisons, we over-sampled the urban population (25%). Addis Ababa held close to 50% of the urban population of Ethiopia [22]; therefore we aimed to take half the urban sample there and the remainder equally from the other three selected Regions. Rural respondents came equally from the latter three Regions.

### Selection and training of interviewers

In Addis Ababa, the interviewer was a nurse from the Department of Neurology, Addis Ababa University, College of Health Sciences. In the other three Regions, interviewers were nurses from health centres in urban areas and Health Extension Workers from health stations in rural areas (the latter are young women with at least grade-10 education and one year of didactic and practical training in health care; their primary charge is to promote health through education, screening, prevention and selective clinical interventions [23]). All interviewers attended a 3-day training session at their respective health-service facilities, including clinical aspects of headache disorders and the theoretical and practical aspects of the study design and purpose, and were then assessed in supervised interviews.

### Questionnaire

We used a culturally modified version of the structured Headache-Attributed Restriction, Disability, Social Handicap and Impaired Participation (HARDSHIP) questionnaire [2], translated according to LTB’s translation protocol for lay documents [24] into two local languages: Oromo for the Oromia Region and Amharic for the remaining three Regions. We piloted the questionnaire for acceptability first in Addis Ababa in the original English, translated at point of application by the interviewer, then in the translated versions in a total sample of 160 from all Regions (40 urban, 120 rural), using a mixture of convenience and purposive sampling. We modified questions accordingly. These participants were not included in the study results.

The questionnaire was composed of five parts: personal and demographic enquiry, and headache screening questions, which were addressed to all respondents; these were followed in those screening positively by diagnostic questions based on ICHD-II [25], enquiry into burden and questions on selected comorbidities. The screening question for headache was: “In the last year, have you had headache?” Participants who answered “no” were classified as headache-free; those who answered “yes” were asked if all their headaches were of one or more types and, if more than one, to focus in the subsequent questions on the one that was most bothersome. The point prevalence of headache was estimated by asking: “Did you have a headache yesterday?”

### Main study

This was completed during April and May 2014.

In each Region, Ethiopian nationals aged 18–65 years were randomly sampled by cold-calling door-to-door. We randomly selected a zone (study area) within each Region and a district within each zone. The sampling procedure was designed to accommodate multiple families living in single dwelling-places in unplanned settlements. In each district the interviewer randomly selected a block or circumscribed area of dwellings and, within that block or area, one or more dwellings. In each selected dwelling, the interviewer first identified the number of families living there, in one or more households (defined as sharing a kitchen). Each biologically unrelated family within a dwelling was included, but only one participant was selected from each family. Where a member of one family in a dwelling was a first- or second-degree relative of a member of another family also within that dwelling, the two families were considered as one.

The female head of each family was asked to list all adult family members living within that dwelling. From this list, one person was randomly selected. Only this person was included in the sample. If present, he or she was immediately asked the demographic and screening questions; if not, the female head-of-family answered these questions on behalf of the participant. In either case, if the screening question was answered negatively the interview was complete at that point. If the screening question was answered positively, and the selected respondent was present, the full interview continued; if the selected respondent was not present, an appointment was made for the interviewer to return for this purpose.

Where the door to a selected dwelling was not answered at first visit, two further attempts were made before the dwelling was excluded and replaced by another according to the sampling algorithm.

Every selected respondent was included unless withholding consent, failing to be available on two occasions for which an appointment had been made, or unable to comprehend the questions. As the survey proceeded, DKW checked each questionnaire and discarded those that were incomplete for essential information. Excluded respondents were replaced from other households, since sampling was continued until the requisite number was achieved.

### Diagnosis

Interviewers did not make diagnoses: these were derived algorithmically during analysis [2]. We first separated participants reporting headache on ≥15 days/month; we described these as a separate group, because they cannot be fully diagnosed by questionnaire. Those among this group who also reported regular use of headache medication on >3 days/week were considered to have pMOH. To all others, the algorithm applied ICHD-II criteria in the order: migraine, TTH, probable migraine, probable TTH [1, 25]. Cases of migraine and probable migraine, and of TTH and probable TTH, were combined for prevalence estimation and further analyses. Remaining cases were unclassified.

### Quality control

To ensure completeness of data collection, each interviewer checked responses to questionnaires before ending each visit. The principal investigator made random unannounced checks, in the field, of the interviewers’ work-quality. The interviewers were told that this might occur at any time.

### Data entry and management

Data from questionnaires were entered into a secure database. Before entry, the forms were scrutinised for accuracy, completeness, inconsistencies, wrong entries, illegible markings and missed entries. Two epidemiologists from Addis Ababa University entered the data independently, with cross-checking. Mismatches were fewer than 0.5%, and resolved by referring to the original forms.

### Statistics and analysis

We aimed for a sample size of 2,000 according to published guidelines [26]. For planning purposes we anticipated a 20% non-participation proportion and a requirement to visit 2,400 households.

We analysed age as a categorical variable (18–25, 26–35, 36–45, 46–55, 56–65 years). We classified marital status as single, married, widowed or divorced, educational level as illiterate (none), primary (grades 1–7), secondary (grades 8–12) or higher (college or university), employment status as unemployed or as unskilled, skilled or professional work. We categorised income per capita per month as ≤ ETB 500 or > ETB 500 (at the time of the survey, USD 1.00 = ETB 18.93, so this was approximately USD 1.00/day).

We used descriptive summaries for sociodemographic and clinical characteristics. We estimated prevalences as proportions (%). We measured headache frequency in days/month, multiplying by 12 to estimate days/year, and expressed this as a mean ± standard deviation (SD). We used chi-squared and Fisher’s exact tests in comparisons of proportions and distributions. We used logistic regression analysis to examine associations between demographic variables and prevalence, calculating odds ratios (ORs) with 95% confidence intervals (CIs). We regarded *p* < 0.05 as significant.

Analyses were performed using SPSS/PC version 20.0 software packages for statistical analysis (SPSS, INC, Chicago, IL) and Excel Professional Plus 2010 Version 14.0.7166.5000.

## Results

From 2,528 households approached, 2,461 biologically unrelated participants were successfully interviewed of whom 2,385 met the entry criteria and were included in the analysis (exclusions were aged >65 years). There were 61 household refusals in the urban study area and none in the rural areas; these were not counted as non-participants since we had not established the presence of anyone eligible [1]. There were 6 refusals by selected respondents, 2 (0.3%) in the urban area and 4 (0.2%) in the rural areas. The overall participating proportion was 99.8%.

The sociodemographic characteristics of participants are displayed in Table 1. Females (55.7%) outnumbered males (44.3%); the expected ratio was 50.3:49.7 [21] (chi-squared = 27.628; *p* < 0.0001). The age distribution of our sample reasonably matched that of the national population except for under-representation in the 18–25 age group (estimated expected proportion 27% based on 2011 data [21]; chi-squared = 7.903; *p* = 0.0049), which occurred in the rural sample. Because we deliberately over-sampled the urban population, our sample (25:75) did not match the national urban/rural distribution (17:83 [21]; chi-squared = 107.895; *p* < 0.0001). Adjustments to observed prevalences were therefore necessary for habitation as well as age and gender, which, accordingly, are reported below. Rural participants were not only significantly older than urban (*p* < 0.0001) but also poorer (*p* <0.0001) and less well educated (*p* < 0.0001), with almost half (49.0%) being illiterate.

**Table 1:** Demographic characteristics of the study sample

	All n (%)	Rural n (%)	Urban n (%)	*p* ^a^
Total	2,385	1,789 (75.0)	596 (25.0)	–
Gender				
Female	1,328 (55.7)	970 (54.2)	358 (60.1)	
Male	1,057 (44.3)	819 (45.8)	238 (39.9)	0.0133^b^
Age (years)				
18–25	583 (24.4)	399 (22.3)	184 (30.9)	<0.0001^c^
26–35	873 (36.7)	648 (36.2)	225 (37.8)	
36–45	452 (19.0)	351 (19.6)	101 (16.9)	
46–55	325 (13.6)	264 (14.8)	61 (10.2)	
56–65	152 (6.4)	127 (7.1)	25 (4.2)	
Marital status				
Single	1,597 (67.0)	1,317 (73.6)	280 (47.0)	<0.0001^c^
Married	480 (20.1)	239 (13.4)	241 (40.4)	
Widowed	104 (4.4)	63 (3.5)	41 (6.9)	
Divorced	204 (8.6)	170 (9.5)	34 (5.7)	
Educational level				
None (illiterate)	947 (39.7)	876 (49.0)	71 (11.9)	<0.0001^c^
Primary	756 (31.7)	602 (33.7)	154 (25.8)	
Secondary	449 (18.8)	235 (13.1)	214 (35.9)	
Higher	233 (9.8)	76 (4.2)	157 (26.3)	
Employment				
Housewife	829 (34.8)	723 (40.4)	106 (17.8)	<0.0001^c^
Student	219 (9.2)	141 (7.9)	78 (13.1)	
Employed (non-farmer)	598 (25.1)	230 (12.9)	368 (61.7)	
Farmer	660 (27.7)	659 (36.8)	1 (0.2)	
Unemployed	47 (2.0)	8 (0.4)	39 (6.5)	
Retired	32 (1.3)	28 (1.6)	4 (0.7)	
Income per month (ETB)^d^				
≤ 500	958 (40.5)	887 (49.7)	71 (12.3)	<0.0001^b^
> 500	1,405 (59.5)	897 (50.3)	508 (87.7)	

^a^comparing distributions between rural and urban participants; ^b^Fisher’s exact test; ^c^chi-squared test; ^d^results missing for 22 participants; ETB: Ethiopian birr (at the time of the study, USD 1.00 = ETB 18.93; therefore ETB 500/month was approximately USD 1/day)

Prevalence overall and by age, gender and habitation is set out in Table 2. In total, 1,071 participants (44.9% [95% CI: 42.4–46.3]; males 37.7%, females 49.9%) reported headache in the preceding year. Table 2 shows both the observed prevalences of each headache type, and adjusted values for gender, age and habitation. TTH was the most prevalent (observed: 20.7% [definite: 15.5%; probable: 5.2%]; adjusted: 20.6%), slightly ahead of migraine (observed: 19.0% [definite 10.8%; probable 8.2%]; adjusted: 17.7%). All causes of headache on ≥15 days/month were 3.2% and pMOH 0.7% (both adjusted). There were 38 cases (1.6%) of unclassified headache. Headache on the day prior to the interview (headache yesterday) was reported by 170 participants (7.1% [6.2–8.2]; males 45 [4.1%], females 125 [9.2%]; prevalence adjusted for gender, age and habitation: 6.4%).

**Table 2:** Observed 1-year prevalence (% [95% confidence intervals]) by gender, age and habitation, overall and by headache type, and adjusted values for gender, age and habitation

	Migraine (*n* = 452)	TTH (*n* = 493)	pMOH (*n* = 21)	Other headache on ≥15d/m (*n* = 67)	Any headache yesterday (*n* = 170)
All (*N* = 2,385)	19.0 [17.4–20.6]	20.7 [19.1–22.3]	0.9 [0.5–1.3]	2.8 [2.2–3.6]	7.1 [6.2–8.2]
Gender					
Male (*n* = 1,057)	14.1 [12.0–16.2]	19.8 [17.4–22.2]	0.6 [0.2–1.3]	2.1 [1.4–3.2]	4.1 [2.9–5.3]
Female (*n* = 1,328)	22.8 [20.5–25.1]	21.4 [19.2–23–6]	1.1 [0.7–1.9]	3.4 [2.5–4.5]	9.2 [7.7–10.8]
Age (yr)					
18–25 (*n* = 583)	18.4 [15.3–21.6]	16.0 [13.0–19.0]	1.0 [0.4–2.3]	2.1 [1.1–3.6]	6.2 [4.5–8.5]
26–35 (*n* = 873)	20.4 [17.7–23.1]	20.8 [18.1–23.5]	0.7 [0.3–1.2]	1.9 [1.0–2.8]	6.8 [5.3–8.6]
36–45 (*n* = 452)	18.1 [14.6–21.7]	23.9 [20.0–27.8]	0.9 [0.3–2.3]	2.2 [0.9–3.6]	8.2 [6.0–11.1]
46–55 (*n* = 325)	19.4 [15.1–23.7]	19.1 [14.8–23.4]	1.5 [0.6–3.7]	5.2 [3.2–8.3]	8.6 [6.0–12.2]
56–65 (*n* = 152)	14.5 [8.9–20.1]	31.6 [24.2–39.0]	1.3 [0.1–5.0]	2.6 [0.8–6.8]	6.6 [3.5–11.8]
Habitation					
Rural (*n* = 1,789)	18.6 [16.8–20.4]	24.4 [22.4–26.4]	0.4 [0.2–0.8]	2.7 [2.0–3.6]	6.7 [5.6–7.9]
Urban (*n* = 596)	20.0 [16.8–23.2]	9.4 [7.1–11.7]	2.4 [1.4–3.9]	3.2 [2.0–5.0]	8.6 [6.6–11.1]
Adjusted for gender, age and habitation	17.7%	20.6%	0.7%	2.5%	6.4%

*TTH* tension-type headache, *pMOH* probable medication-overuse headache, *d*/*m*: days/month

All headache types were more common in females, migraine by about 3:2 (*p* < 0.0001 [Fisher’s exact]). For TTH (*p* = 0.3596), pMOH (by about 2:1; *p* = 0.1863) and other headache on ≥15 days/month (also by about 3:2; *p* = 0.0613), differences were not significant. Headache yesterday was reported more than twice as commonly by females (*p* < 0.0001). Age relationships were generally rather flat, although pMOH and other headache on ≥15 days/month tended to become more prevalent with advancing age, and this was reflected in headache yesterday. TTH was most common (31.6%) in the oldest age group (56–65 years) and least common (16.0%) in the youngest (≤25 years). TTH was more common in rural areas (*p* < 0.0001), but the opposite was true of pMOH (*p* < 0.0001) and numerically of other headache on ≥15 days/month (*p* = 0.5667); consequently, headache yesterday was reported more often by urban than rural dwellers, although the difference was not significant (*p* = 0.1189).

We used logistic regression analysis to look at associations of migraine, TTH and pMOH with habitation and socioeconomic indicators (Table 3). This analysis confirmed a strong negative association between urban dwelling and TTH (OR: 0.3; *p* < 0.0001), but a very powerful positive association (OR: 6.1; *p* < 0.0001) of urban dwelling with pMOH. Education to any level was associated with reduced migraine prevalence, and above primary level with reduced TTH prevalence; however, education to secondary level and especially above (OR: 2.9; *p* = 0.067) appeared to raise the odds of pMOH, although these associations were not significant. These findings were in line with associations with income: migraine was less prevalent (OR: 0.8; *p* = 0.035) and pMOH more prevalent (OR: 2.1; not significant) in those with incomes above ETB 500/month.

**Table 3:** Logistic regression analysis of associations with each diagnosis adjusting for gender, age and all variables listed

	Migraine		Tension-type headache		pMOH	
	OR	*p*	OR	*p*	OR	*p*
Habitation						
Rural	reference		reference		reference	
Urban	1.1 [0.9–1.4]	0.466	0.3 [0.2–0.4]	<0.0001	6.1 [2.5–15.3]	<0.0001
Education						
None	reference		reference		reference	
Primary	0.5 [0.4–0.6]	<0.0001	1.0 [0.8–1.3]	0.908	0.5 [0.1–2.1]	0.367
Secondary	0.6 [0.4–0.8]	0.000	0.6 [0.5–0.8]	0.003	1.8 [0.6–5.5]	0.284
College or University	0.7 [0.5–1.0]	0.042	0.4 [0.4–0.8]	0.006	2.9 [0.9–9.4]	0.067
Employment						
Employed (non-farming)	reference		reference		numbers too small to calculate	
Housewife	2.8 [2.1–3.7]	<0.0001	3.0 [2.2–4.2]	<0.0001		
Student	0.3 [0.2–0.4]	<0.0001	0.4 [0.2–0.6]	<0.0001		
Farmer	1.2 [0.9–1.7]	0.159	2.7 [2.0–3.8]	<0.0001		
Unemployed	0.05 [0.0–0.1]	<0.0001	0.05 [0.02–0.1]	<0.0001		
Retired	0.02 [0.001–0.1]	<0.0001	0.2 [0.1–0.3]	<0.0001		
Income per month (ETB)						
≤ 500	reference		reference		reference	
> 500	0.8 [0.7–1.0]	0.035	1.0 [0.8–1.2]	0.806	2.1 [.0.7–5.7]	0.164

*pMOH* probable medication-overuse headache, *OR* odds ratio with 95% confidence interval in parenthesis, *ETB* Ethiopian birr (at the time of the study, USD 1.00 = ETB 18.93; therefore ETB 500/month was approximately USD 1/day)

## Discussion

This study was the first population-based survey using established methodology [1] and questionnaire [2] to estimate the prevalence of primary headache disorders in Ethiopia. Minimising potential biases, it included diverse regions, employed an appropriate mix of cluster-sampling and simple random sampling, applied ICHD-II diagnostic criteria [25] and had a very high participating proportion (99.8%). It found that headache disorders were highly prevalent in this country: 1-year prevalence of any headache was 44.9%; adjusted for gender, age and habitation (urban/rural), the 1-year prevalence of migraine was 17.7%, of TTH 20.6%, of all causes of headache on ≥15 days/month 3.2%, and of pMOH 0.7%. There was a very small proportion (1.6%) of unclassified headache. TTH (OR: 0.3) was less prevalent among the urban population, but urban dwelling was very strongly associated (OR: 6.1) with pMOH. Education was associated with reduced migraine prevalence (OR: 0.5–0.7) but a tendency to raised odds of pMOH (OR: 2.9 [*p* = 0.067] at university level). Income above ETB 500/month (approximately USD 1/day) showed associations in expected alignment with these.

This was the second such study within the Global Campaign against Headache to be conducted in SSA: the first was in Zambia, and showed not dissimilar overall results with regard to both migraine (gender- and habitation-adjusted 1-year prevalence 22.9%) and TTH (22.8%) [17]. TTH was the most prevalent headache in Ethiopia, but only by a small margin, and not so in Zambia [17]; we speculate that this may have been due to cultural under-reporting of infrequent episodic TTH in both countries. These findings confirm that, contrary to earlier estimates from Benin [12], Tanzania [8, 11, 13] and, indeed, Ethiopia [7, 27]), primary headache is at least as common in SSA as in the rest of the world [16]. In Zambia, TTH was less common among the university-educated (OR: 0.22) but migraine was more prevalent (OR: 2.1) [17]. However, the principal difference between the countries was in pMOH: 0.7% in Ethiopia, 7.1% in Zambia [17]. Nevertheless, in Ethiopia as in Zambia, pMOH was very much a problem in the urban population rather than rural (Zambia is far more urbanised [40%] [28] than Ethiopia [17%] [21]).

Migraine prevalence in an Ethiopian rural community was previously estimated at only 3% [27], far lower than our 17.7% for an urban/rural mix. In the earlier study, the focus was on chronic recurrent headache (with a reported overall prevalence of 4.7%), which might attract priority in the context of the other challenges of daily life in rural Ethiopia. The screening question was “Do you have recurrent headache”, and the survey stopped when participants answered “No” to this question. It is highly likely that people with occasional headache (the majority with headache) were excluded. In addition, the diagnostic questionnaire would diagnose migraine only when all ICHD-I criteria were met (*ie*, excluding probable migraine). These factors together would sufficiently explain the difference between 3 and 17.7%.

Headache yesterday was reported much less frequently in Ethiopia (6.4% adjusted) than Zambia (19.1% adjusted [17, 18]). The reasons are clear: not only pMOH but all causes of headache on ≥15 days/month (key drivers of headache yesterday) were much more common in Zambia, and highly associated with urban dwelling in a more urbanised population. Headache yesterday is equivalent to 1-day prevalence, and can be predicted from the 1-year prevalence of all headache (45.2%) and mean headache frequency in days/month in those affected (4.2). The latter divided by 30 gives the probability of headache on any particular day among those with headache (in this case 0.14); predicted 1-day prevalence is the product of this and 1-year prevalence (*ie*, 6.3%). The observed prevalence of headache yesterday (7.1%) was close to this, which affirms the veracity of these findings. It also demonstrates one benefit of epidemiological enquiry into headache yesterday.

The strengths of this study have been mentioned above. Its principal limitation was that we surveyed in only two languages, while there are over 80 active languages in Ethiopia [29]. Oromo and Amharic are the most widely used: according to 2014 census data, Oromo is spoken by 33.8% of the total population, Amharic by 29.3% [29]. This suggests we can claim to represent a majority (63%) but not all of the population. The logistic difficulties and resource implications associated with each additional language would not have justified including even one more (other key languages: Somali [6.3%], Tigrinya [5.9%], Sidamo [4.8%], Wolaytta [2.2%], Gurage [2.0%] and Afar [1.7%] [29]). We also could not validate the diagnostic questionnaire in the two languages. This would have required at least one of the very few headache experts in the country to take time out from the health service in order to re-interview a subset of participants in the field; we could not justify this. The questionnaire already had a record of successful use in many countries and cultures [2, 17, 18, 30–35].

What are the implications of these findings? For Ethiopia, they show that, far from being relatively uncommon, as past research has indicated, headache disorders are highly prevalent. Since headache disorders are the third greatest cause of disability globally [5, 6], with migraine the principal contributor and MOH also important, it is very probable that they are also in Ethiopia. Data are needed to confirm this, and will be reported in a future publication. However, migraine prevalence in Ethiopia (17.7%) is 20% higher than the estimated global mean reported in GBD 2010 (14.7%) [36]. While pMOH is less common overall (0.7%) than the estimated global mean (about 1.5% [37]), its prevalence in the Ethiopian urban population (2.4%) exceeds it by about 60%. MOH is the result of a form of mistreatment. It is fostered wherever limited access to health care, and limited expertise in management of headache disorders among the health-care providers who are available, lead to a culture, unrestrained by effective public health-education, of recourse to analgesics obtained over-the-counter (OTC). Escalating use is then a behaviour typically leading to MOH everywhere. However, this is obviously dependent on ready access to OTC drugs – both availability, and the means to buy them. It is this, almost certainly, that explains why pMOH is selectively an urban problem, here in Ethiopia as it is in Zambia [17]. Whatever health care is provided for headache in Ethiopia, public education regarding the risk of MOH is needed, and could be provided to the urban population at relatively low cost [38].

There are also implications for the Global Burden of Disease project itself [20]. SSA is a large and populous area of the world: as population-based data from SSA increasingly convincingly demonstrate that headache disorders are very common here, and are incorporated into future iterations of GBD, estimates of headache-attributed burden will rise not only in SSA but globally.

## Conclusions

Findings for migraine and TTH in Ethiopia, showing high prevalence of the former, were quite similar to those from Zambia, another SSA country. While pMOH was much less prevalent, it was as in Zambia essentially an urban problem. Primary headache disorders are at least as common in Ethiopia as in high-income western countries, and this study offers confirmation that this is true of the SSA region.

## Abbreviations

CI: Confidence interval
d/m: days/month
ETB: Ethiopian birr
GBD: Global Burden of Disease
HARDSHIP: Headache-Attributed Restriction, Disability, Social Handicap and Impaired Participation questionnaire
ICHD: International Classification of Headache Disorders
LTB: *Lifting The Burden*
MOH: Medication-overuse headache
OR: Odds ratio
pMOH: Probable MOH
SD: Standard deviation
SNNPRS: South Nations Nationalities and People’s Regions States
SSA: Sub-Saharan Africa
TTH: Tension-type headache
USD: United States dollar
WHO: World Health Organization
YLD: Year of life lost to disability
